# Integrating Genetic Alterations and the Hippo Pathway in Head and Neck Squamous Cell Carcinoma for Future Precision Medicine

**DOI:** 10.3390/jpm12101544

**Published:** 2022-09-20

**Authors:** Toshinori Ando, Kento Okamoto, Tomoaki Shintani, Souichi Yanamoto, Mutsumi Miyauchi, J. Silvio Gutkind, Mikihito Kajiya

**Affiliations:** 1 Center of Oral Clinical Examination, Hiroshima University Hospital, Hiroshima 734-8551, Japan; 2 Department of Oral Oncology, Graduate School of Biomedical and Health Sciences, Hiroshima University, Hiroshima 734-8553, Japan; 3 Department of Oral and Maxillofacial Pathobiology, Graduate School of Biomedical and Health Sciences, Hiroshima University, Hiroshima 734-8553, Japan; 4 Moores Cancer Center, University of California, San Diego, CA 92093, USA; 5 Department of Pharmacology, University of California, San Diego, CA 92093, USA

**Keywords:** Hippo pathway, YAP/TAZ, genetic alterations, HNSCC

## Abstract

Genetic alterations and dysregulation of signaling pathways are indispensable for the initiation and progression of cancer. Understanding the genetic, molecular, and signaling diversities in cancer patients has driven a dynamic change in cancer therapy. Patients can select a suitable molecularly targeted therapy or immune checkpoint inhibitor based on the driver gene alterations determined by sequencing of cancer tissue. This “precision medicine” approach requires detailed elucidation of the mechanisms connecting genetic alterations of driver genes and aberrant downstream signaling pathways. The regulatory mechanisms of the Hippo pathway and Yes-associated protein/transcriptional co-activator with PDZ binding motif (YAP/TAZ) that have central roles in cancer cell proliferation are not fully understood, reflecting their recent discovery. Nevertheless, emerging evidence has shown that various genetic alterations dysregulate the Hippo pathway and hyperactivate YAP/TAZ in cancers, including head and neck squamous cell carcinoma (HNSCC). Here, we summarize the latest evidence linking genetic alterations and the Hippo pathway in HNSCC, with the aim of contributing to the continued development of precision medicine.

## 1. Introduction

**Figure 1:**
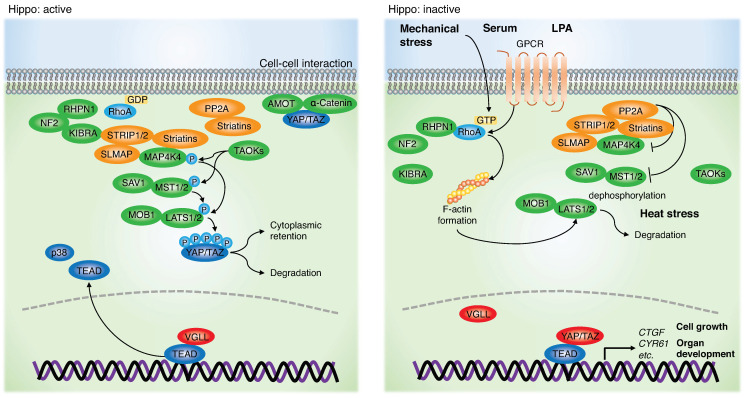
Roles of the Hippo pathway and YAP/TAZ. TAOKs phosphorylate MAP4Ks and MST1/2. Activated MAP4Ks and MST1/2 phosphorylate LATS1/2. Activated LATS1/2 phosphorylates YAP/TAZ at multiple serine residues, leading to its cytoplasmic retention or degradation. Cell-cell interaction induces cytoplasmic sequestration of YAP/TAZ by AMOT and α-catenin. While the Hippo is inactive, dephosphorylated YAP/TAZ translocates to the nucleus, binds to TEAD, and enhances transcription of growth-related genes. Mechanical stress induces RhoA activation and F-actin formation, thereby inhibiting LATS1/2. Serum or LPA treatment inhibits MAP4K4 and MST1/2 through STRIPAK formation, which causes LATS1/2 inactivation. Heat stress induces LATS1/2 dephosphorylation and degradation. Abbreviations are: ras homolog family member A (RhoA), Rho GTPase binding protein 1 (RHPN1), Neurofibromin 2 (NF2; also known as Merlin), kidney and brain protein (KIBRA; also known as WWC1), STRN-interacting protein (STRIP1/2), sarcolemmal membrane-associated protein (SLMAP), the angiomotin (AMOT) family, Mitogen-activated protein kinase kinase kinase kinase 4 (MAP4K4), TAO kinases (TAOKs), mammalian STE20-like kinase 1 and 2 (MST1/2; MST2 is also known as STK3), salvador homolog 1 (SAV1), large tumor suppressor 1 and 2 (LATS1/2), MOB kinase activator 1A and 1B (MOB1A/B), Yes-associated protein (YAP), and transcriptional co-activator with PDZ binding motif (TAZ; also known as WWTR1), ras homolog family member A (RhoA), vestigial-like proteins (VGLL), TEA domain family members (TEAD), *connective growth factor* (*CTGF*), and *cysteine-rich angiogenic inducer 61* (*CYR61*), lysophosphatidic acid (LPA), and G protein-coupled receptor (GPCR).

The Hippo pathway is a tumor-suppressive signaling axis. Its downstream effectors, Yes-associated protein (YAP) and transcriptional co-activator with PDZ binding motif (TAZ), are essential in normal cell growth, tissue growth, and organ size [1]. In mammals, the core Hippo kinase pathway consists of mammalian STE20-like kinase 1 and 2 (MST1/2), large tumor suppressor 1 and 2 (LATS1/2), and their respective adaptor proteins, Salvador homolog 1 (SAV1) and MOB kinase activators 1A and 1B (MOB1A/B) [2,3,4,5,6,7,8,9]. When the Hippo pathway is active, MST1/2 phosphorylates LATS1/2. This, in turn, activates LATS1/2 phosphorylation of YAP/TAZ at five serine residues, resulting in their cytoplasmic retention by binding to 14-3-3 and/or degradation by the ubiquitin-proteasome pathway [10]. In contrast, when the Hippo pathway is inactive, hypophosphorylated YAP/TAZ translocates into the nucleus and interacts with transcription factors, including TEA domain family members (TEAD), to promote the transcription of growth-related genes, including *connective growth factor* (*CTGF*) and *cysteine-rich angiogenic inducer 61* (*CYR61*) [11]. Similar to YAP/TAZ, vestigial-like proteins 1-4 (VGLL1-4) also bind to TEAD through their Tondu (TDU) domains. VGLL1-3 contains only one TDU domain at its C-terminus, while VGLL4 has two TDU domains [12,13,14,15]. The function of VGLL is still poorly understood. VGLL1 promotes proliferation of prostate cancer cells [14]. However, VGLL2-4, especially VGLL4, transcriptionally suppress TEAD by competing with YAP [16,17,18,19] (Figure 1).

In addition to the main Hippo components (MST1/2, LATS1/2, SAV1, and MOB1), multiple molecules have been identified as regulators of the Hippo pathway. The angiomotin (AMOT) family, neurofibromin 2 (NF2; also known as Merlin), kidney and brain protein (KIBRA; also known as WWC1), FERM domain-containing 6 (FRMD6), LIM domain-containing protein AJUBA, zonula occludens, and α-catenin are involved in cell-cell junctions and regulate the Hippo pathway [20]. Therefore, the loss of cell-cell adhesion inactivates the Hippo pathway and activates YAP/TAZ [21]. Mitogen-activated protein kinase kinase kinase kinase (MAP4K) family members consist of hematopoietic progenitor kinase 1 (HPK1/MAP4K1), germinal center kinase (GCK/MAP4K2), germinal center kinase-like kinase (GLK/MAP4K3), HPK/GCK-like kinase (HGK/MAP4K4), misshapen-like kinase 1 (MINK1/MAP4K6), and TRAF2 and NCK interacting kinase (TNIK/MAP4K7). These enzymes can phosphorylate the hydrophobic motif (T1079) of LATS1/2, leading to YAP inactivation [22]. TAO kinases (TAOKs) phosphorylate MST1/2 and MAP4Ks, inducing their activation [23,24,25]. Striatin (STRN)-interacting phosphatase and kinase (STRIPAK) is a protein complex composed of PP2A catalytic subunit (PP2AC), scaffolding subunit (PP2AA), and the STRN regulatory subunit. The complex recruits STRN-interacting protein (STRIP1/2), sarcolemmal membrane-associated protein (SLMAP), tumor necrosis factor receptor-associated factor 3-interacting protein 3 (TRAF3IP3), suppressor of IKBKE1 (SIKE1), fibroblast growth factor receptor 1 oncogene partner 2 (FGFR1OP2), cortactin-binding protein 2 (CTTNBP2), CTTNBP2 N-terminal-like protein (CTTNBP2NL), MOB4, GCK family, and cerebral cavernous malformations 3 (CCM3) [26]. Serum or lysophosphatidic acid (LPA) stimulation activates ras homolog family member A (RhoA) and dissociates rhophilin Rho GTPase binding protein 1 (RHPN1) and NF2/KIBRA from STRIPAK, inducing the dephosphorylation of MST1/2 and MAP4K4 by STRIPAK [27]. STRIPAK induces dephosphorylation of MAP4K4, leading to Hippo pathway inactivation and YAP activation [28]. Heat stress induces dephosphorylation and degradation of LATS1/2 by heat shock protein 90 (HSP90) and protein phosphatase 5 (PP5) [29]. Through these aforementioned multiple regulators, the Hippo pathway and YAP/TAZ are controlled by physiological conditions including cell density, mechanical stress, serum through G protein-coupled receptors, and heat stress [29,30] (Figure 1).

The Hippo pathway is dysregulated and YAP/TAZ is predominantly hyperactivated in multiple types of cancer [31]. Genetic alterations are strongly involved in dysregulation of the Hippo pathway. For example, NF2, a suppressor of the Hippo pathway, is frequently mutated or inactivated by gene copy number loss, which leads to the activation of YAP/TAZ in meningiomas, mesotheliomas, and peripheral nerve sheath tumors [31]. *GNAQ* or *GNA11*, encoding Gαq and Gα_11_, respectively, are frequently mutated in uveal melanomas (>80%), which causes YAP/TAZ hyperactivation, resulting in uveal melanoma development [32,33]. Liver kinase B1 (LKB1; also known as STK11) is frequently mutated in non-small-cell lung carcinomas (15–35%) and cervical carcinomas (20%) [34]. LKB1 regulates the microtubule affinity-regulating kinase (MARK) family and scribble homolog (SCRIB) to activate MST1/2 and LATS1/2 [35]. These findings suggest that the somatic mutation of LKB1 drives carcinogenesis through YAP/TAZ activation. In terms of the treatment, however, no drug targeting the Hippo-YAP/TAZ pathway is approved by the Food and Drug Administration (FDA) for the treatment of any types of cancer. Strikingly, despite the oncogenic role of YAP/TAZ in most cancers, the function of the Hippo pathway and YAP/TAZ depends on cancer type. YAP/TAZ plays a tumor suppressive role by inducing integrin alpha 5 and beta 5 in neural/neuroendocrine and *RB transcriptional corepressor 1* (*RB1)*^−/−^ solid cancers, such as retinoblastoma, small cell lung cancer, and neuroendocrine prostate cancer [36]. In estrogen receptor-alpha positive (ERα^+^) breast cancer, LATS1/2 regulates ERα expression. Loss of LATS1/2 selectively suppresses the proliferation of ERα^+^, but not ERα^−^, breast cancer cells [37]. Moreover, activated YAP and TEAD enhance VGLL3 transcription. In turn, VGLL3 interacts with TEAD and NCOR2 and inhibits *ESR1* transcription from its super enhancer region [38]. LATS1/2 has an oncogenic role by sustaining the Wnt pathway and stem cell function in the intestine, thereby promoting colorectal malignancies [39]. Therefore, cancer type-specific roles of YAP/TAZ should be carefully considered and investigated for precision medicine targeting the Hippo and YAP/TAZ pathways.

Head and neck squamous cell carcinoma (HNSCC) is diagnosed in approximately 54,000 new cases each year in the United States alone, resulting in more than 11,230 deaths [40]. The Cancer Genome Atlas (TCGA) network contains comprehensive information regarding gene alterations in HNSCC. This data have accelerated the understanding of the connections between gene alterations and Hippo pathway dysregulation [41]. Emerging evidence has linked genetic alterations with Hippo pathway dysregulation and aberrant YAP/TAZ activation, which are essential for HNSCC initiation and progression. Here, we introduce the latest discoveries connecting genetic alterations and the Hippo pathway in HNSCC. The aim of this review is to assist the further development of precision medicines for HNSCC patients.

## 2. Role of the Hippo Pathway and YAP/TAZ in HNSCC

YAP/TAZ protein levels are generally sustained at low levels in the prickled layer but are higher in the basal cells in the normal mucosal epithelium of the head and neck region. YAP/TAZ protein levels increase in precancerous and invasive HNSCC [42]. Nuclear YAP activation is more evident in poorly differentiated HNSCC than in well-differentiated HNSCC [43]. Conditional MOB1A/B double knockout in basal cells initiates squamous cell carcinoma of the tongue within four weeks, suggesting that YAP hyperactivation is sufficient for HNSCC initiation [44]. Several mouse studies have also shown that YAP/TAZ is required for the initiation of basal cell carcinoma and skin squamous cell carcinoma [45,46]. Moreover, loss of heterotrimeric G protein Gα_s_ and protein kinase A (PKA) signaling promotes YAP and glioma-associated oncogene homolog 1 (GLI1) activities, which are sufficient for hair follicle stem cell expansion and basal cell carcinoma initiation [47]. In HNSCC tissue, YAP expression is higher in the invasive front of the tumor than proximal region [48]. Single cell approach revealed partial epithelial-to-mesenchymal transition (EMT) cells localize in the invasive front [49]. Partial-EMT cells also promote cancer stemness [50]. Collectively, these results indicate that YAP/TAZ hyperactivation sustains and expands stem or progenitor cells in the basal cell layer of the mucosal epithelium in the head and neck region, leading to HNSCC initiation and progression.

## 3. Genetic Alterations and Dysregulation of the Hippo Pathway in HNSCC

**Figure 2:**
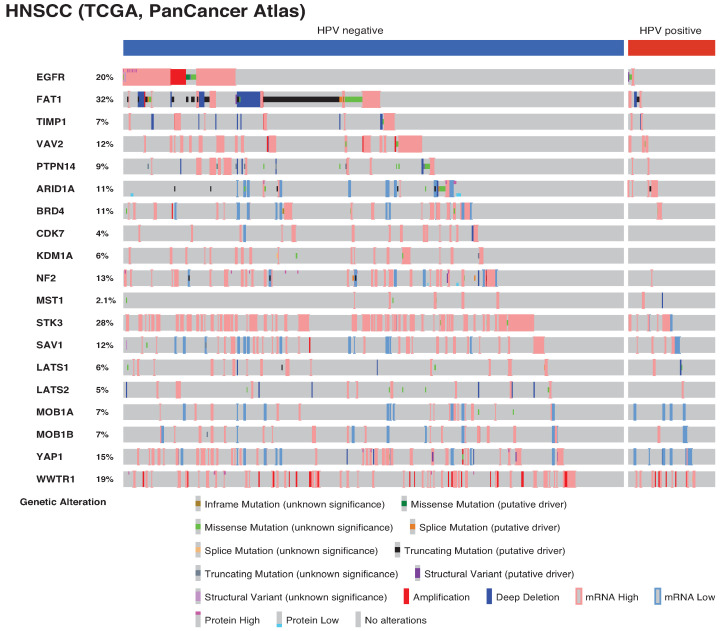
Landscape of the genetic alterations dysregulating the Hippo pathway and YAP/TAZ in HNSCC. The landscape was created by c-BioPortal for Cancer Genomics (https://www.cbioportal.org accessed on 19 September 2022) by analyzing the data of the HNSCC TCGA, PanCancer Atlas (2015). Abbreviations are: Epidermal growth factor receptor (EGFR), FAT atypical cadherin 1 (FAT1), tissue inhibitor of metalloproteinase-1 (TIMP-1), vav guanine nucleotide exchange factor 2 (VAV2), protein tyrosine phosphatase non-receptor type 14 (PTPN14), AT-rich interaction domain 1A (ARID1A), bromodomain containing protein 4 (BRD4), Cyclin dependent kinase 7 (CDK7), and Lysine-specific demethylase 1 (LSD1).

**YAP1 amplification and minor gene alterations in Hippo components.** TCGA includes a comprehensive landscape of somatic genetic alterations in HNSCC, with the second highest incidence of *YAP1* gene amplification (4.4% of the cases) and the third highest incidence of *WWTR1* (*TAZ*) gene amplification (8.8%) among all cancers [51]. However, copy number loss or mutations in the core Hippo components (MST1/2, LATS1/2, SAV1, and MOB1A/B) are rare in HNSCC (Figure 2). This suggests that genetic alterations coding for the molecules surrounding the core Hippo pathway components or interacting with YAP/TAZ/TEADs in the nucleus are more important for Hippo pathway dysregulation, leading to YAP/TAZ hyperactivation.

**Figure 3:**
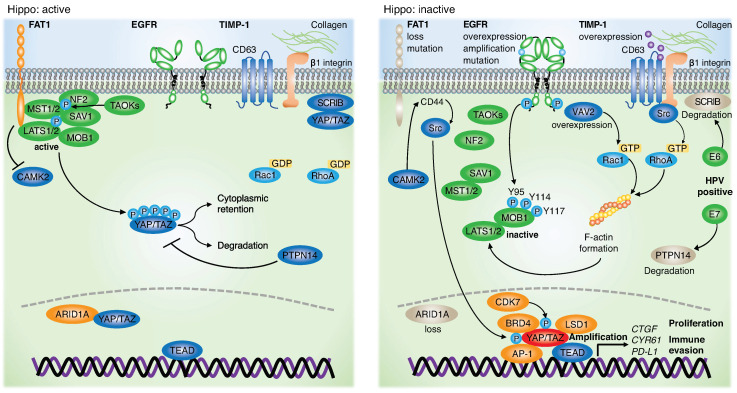
Schematic of the Hippo pathway and YAP/TAZ regulatory components in HNSCC. When the Hippo pathway is active, activated LATS1/2 phosphorylates YAP/TAZ at multiple serine residues, leading to its cytoplasmic retention or degradation. When Hippo is inactive, dephosphorylated YAP/TAZ translocates to the nucleus, binds to TEAD, and enhances transcription of growth-related genes. Genetic alterations including FAT1, EGFR, VAV2, TIMP-1, HPV (E7), ARID1A, BRD4, LSD1, AP-1, CDK7, and YAP1 are involved in aberrant YAP/TAZ activation. Abbreviations are: Rac family small GTPase 1 (RAC1), Ca2+/calmodulin-dependent protein kinase II (CAMK2), and scribble homolog (SCRIB).

**Human papillomavirus (HPV) infection.** HPV is an important subset of HNSCC cases globally [52]. Specifically, HPV-16 and HPV-18 are high-risk subtypes associated with many malignancies, including cervical, head and neck, anal, and vulvar cancers [53]. Among the head and neck areas, the oropharynx is the site most frequently affected by HPV, and its incidence is rising [54,55]. E6 and E7 are specific viral oncoproteins that are essential for carcinogenesis through the inhibition of the tumor suppressor proteins p53 and RB [53]. E6/E7 expressing HNSCC is also associated with mammalian target of rapamycin (mTOR) signaling activation [56]. In addition, recent discoveries indicate that YAP/TAZ activation is required for both HPV-negative and HPV-positive HNSCC initiation and progression. The tumor suppressor protein tyrosine phosphatase non-receptor type 14 (PTPN14) is a tumor suppressor inducing YAP inactivation [57,58,59,60]. E7 binds and degrades PTPN14, thereby activating YAP in basal cells in the stratified squamous epithelium, leading to carcinogenesis [61]. E6 homodimerization induces degradation of SCRIB, thereby promoting YAP/TAZ nuclear localization [62]. E6 suppresses YAP degradation and promotes nuclear YAP localization, thereby increasing growth-related genes, including *amphiregulin* (*AREG*) and *transforming growth factor alpha* (*TGFA*), which encode TGF-α, in cervical cancer [63,64]. Hyperactivation of YAP and E6/E7 synergistically promotes the initiation and progression of cervical cancer in vitro and in vivo [64]. Therefore, YAP/TAZ was hyperactivated in HPV-positive HNSCC cells (Figure 3).

**FAT atypical cadherin 1 (FAT1).** FAT1 is a member of the FAT family. *FAT1* is the second most frequently altered gene (29.8%) after *TP53* (70%) [41]. The FAT1 protein is composed of a large extracellular region with cadherin and epidermal growth factor (EGF)-like repeats, a transmembrane region, and an intracellular region [65]. FAT1 can be proteolyzed, and its intracellular domain is translocated into the nucleus [66]. FAT1 has a tumor-suppressive role by interacting with β-catenin and suppressing its nuclear translocation. Thus, frequent somatic mutations in *FAT1* cause aberrant Wnt pathway activation in HNSCC [67]. In addition, *FAT1* somatic mutations are positively correlated with poor survival of HNSCC patients [68]. Patients harboring truncated *FAT1* have a worse prognosis than wild type patients with HPV-negative HNSCC [69]. Bioinformatics and statistical approaches have revealed that *FAT1* mutation is prevalent in HPV-negative HNSCC and is associated with human epidermal growth factor receptor 3 (HER3) activation and reduced epidermal growth factor receptor (EGFR) expression, suggesting that *FAT1* mutation may confer resistance to EGFR-targeted therapy [70].

Notably, recent emerging evidence has clarified a solid link between *FAT1* gene alterations and the Hippo pathway. FAT1 recruits and assembles Hippo components, including NF2, AMOT, MST1/2, SAV1, LATS1/2, and MOB1A/B, and activates them via TAOKs, resulting in YAP inactivation. Thus, frequent deletion or truncation of *FAT1* disperses Hippo components and leads to aberrant activation of YAP in HNSCC [43]. Loss of FAT1 also induces a hybrid epithelial to mesenchymal transition (EMT) state with stemness and metastasis via activation of the Ca2+/calmodulin-dependent protein kinase II (CAMK2)-CD44-SRC-YAP axis in mouse and human squamous cell carcinoma [71]. Given the frequent gene alterations of *FAT1* in HNSCC, *FAT1* gene alterations explain the prevalent YAP activation in HNSCC (Figure 2 and Figure 3).

**Figure 4:**
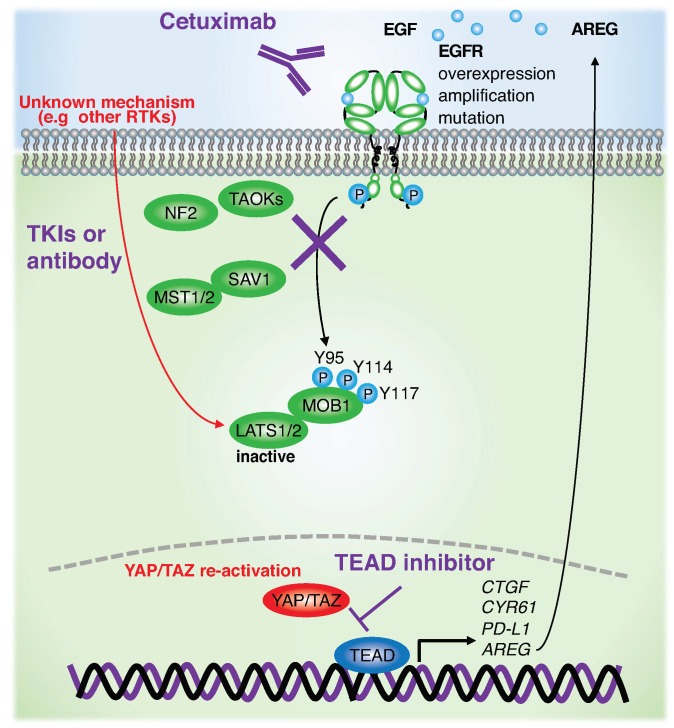
Schematic of the role of Hippo pathway and YAP/TAZ in cetuximab resistance. Cetuximab treatment inactivates EGFR, which in turn reduces tyrosine phosphorylation of MOB1 and activated LATS1/2, resulting in transient YAP/TAZ inactivation. However, unknown mechanisms, including reactivation or overexpression of other receptor types of tyrosine kinases (RTKs), re-activate YAP/TAZ, leading to intrinsic or acquired resistance to cetuximab treatment. Combination therapy with cetuximab and a TEAD inhibitor may be beneficial for avoiding YAP/TAZ reactivation and tumor relapse. AREG denotes amphiregulin (also abbreviated AREG).

**EGFR.** EGFR is an ERBB family tyrosine kinase. EGFR is amplified and highly overexpressed in HNSCC and lung squamous cell carcinoma, frequently mutated and activated in lung adenocarcinoma (LUAC), and mutated, rearranged, and amplified in glioblastoma [41,72,73,74]. Several reports have shown that phosphoinositide-dependent kinase (PDK1) activated by EGFR and phosphoinositide 3-kinase (PI3K) induces Hippo pathway inactivation and YAP activation [75,76]. EGFR, followed by RAS activation, induces SUMOylation of Otubain-2 (OTUB2), thereby promoting deubiquitination of YAP/TAZ and stabilizing them in cancer cells [77]. In addition, recent emerging evidence has revealed that EGFR induces tyrosine phosphorylation of MOB1 at Y95, Y114, and Y117 and inactivates LATS1/2. The resulting activation of YAP/TAZ in HNSCC occurs independent of *FAT1* gene alterations and the PI3K-PDK1 axis [78]. In another study, MOB1A/B double knockout in keratin 14 expressing basal cells resulted in the development of tongue squamous cell carcinoma within 4 weeks, suggesting that MOB1A/B knockout is sufficient to suppress LATS1/2 activity and to hyperactivate YAP/TAZ [44]. Given this evidence, tyrosine phosphorylation of MOB1A/B by EGFR may interfere with the physical interactions between LATS1/2 and MST1/2, MAP4Ks, or TAOKs, resulting in LATS1/2 inactivation. Although EGFR has been targeted by cetuximab, an FDA-approved drug for HNSCC patients, monotherapy response rate remains only 10–30% with intrinsic or acquired resistance [79,80,81]. YAP is amplified and overexpressed in cetuximab-resistant HNSCC cell lines [82]. In LUAC harboring frequent EGFR-activating mutations, EGFR-tyrosine kinase inhibitors (EGFR-TKIs) are the main therapeutic drugs. The emergence of YAP reactivation, EGFR-T790M mutations, or activation of other signaling pathways, including MET proto-oncogene, receptor tyrosine kinase (MET), AXL receptor tyrosine kinase (AXL), insulin-like growth factor 1 receptor (IGF1R), interleukin-6 receptor (IL-6R), human epidermal growth factor receptor 2 (HER2), and HER3, are potentially important for resistance to EGFR-TKIs in LUAC [83,84,85]. The observation that LATS1/2 KO by CRISPR/Cas9 confers resistance to EGFR inhibitors in vitro and in vivo [78] indicate that YAP/TAZ reactivation by an unknown mechanism after EGFR inhibitor treatment plays an important role in HNSCC initial resistance or tumor recurrence. In addition, activated YAP/TAZ promotes transcription and expression of amphiregulin (AREG), which in turn acts as a ligand for EGFR [86]. These findings suggest that the sustained EGFR-YAP-AREG axis represents a positive feedback loop in EGFR-altered cancers. Therefore, targeting YAP/TAZ such as by the use of TEAD inhibitors or inhibitors/antibodies for unknown mechanisms in combination with EGFR-targeting therapy may provide novel therapeutic approaches for preventing cancer recurrence and progression (Figure 2, Figure 3 and Figure 4).

**Mechanotransduction.** Matrix stiffness is an important factor that regulates YAP/TAZ activity. Stiff matrices activate YAP/TAZ through Rho GTPase activity and modification of the actomyosin cytoskeleton [87]. Matrix stiffness activates phospholipase Cγ1 (PLCγ1) to suppress phosphatidylinositol 4,5-bisphosphate (Ptdlns(4,5)P_2_) and phosphatidic acid, which induce Ras-related GTPase RAP2 activation by PDZ domain-containing guanine nucleotide exchange factor 1 (PDZGEF1) and PDZGEF2. At low stiffness, active RAP2 stimulates MAP4K4, MAP4K6, MAP4K7, and ARHGAP29, leading to LATS1/2 activation and YAP/TAZ inactivation [88].

Tissue inhibitor of metalloproteinase-1 (TIMP-1) is a member of the TIMP family (TIMP-1 to -4) that is overexpressed in many cancers, including HNSCC. TIMP-1 high expression in HNSCC is positively correlated with angiogenesis, EMT, metastasis, and worse prognosis [89]. Previous studies have supported the oncogenic role of TIMP-1 in activating the mitogen-activating protein kinase (MAPK) or PI3K-AKT-mTOR pathway independent of its matrix metalloproteinase (MMP) inhibitory function [90,91,92,93,94]. TIMP-1 forms a complex with CD63 and integrin β1 on collagen, and then activates Src and RhoA, resulting in F-actin assembly that inactivates LATS1/2 and activates YAP/TAZ in many types of cancer cell lines, including HNSCC [95,96]. TIMP-1 released from cancer cells promotes the accumulation of cancer-associated fibroblasts (CAFs) within several cancer types [97]. In contrast, CAFs also require YAP/TAZ activation through the activation of Rho-associated, coiled-coil containing protein kinase (ROCK) and Src, which generates a stiffer matrix in cancer tissue [98]. These results suggest that TIMP-1 induces matrix stiffness with fibrosis and that the stiffness simultaneously induces YAP/TAZ activation in cancer cells via mechanotransduction (Figure 2 and Figure 3).

The VAV family (VAV1-3) are guanosine nucleotide exchange factors (GEFs) that are activated through tyrosine phosphorylation by tyrosine kinases [99]. EGFR phosphorylates and activates VAV guanine nucleotide exchange factor 2 (VAV2), resulting in high levels of GTP-bound (activated) Rac family small GTPase 1 (RAC1) in HNSCC [100]. Moreover, VAV2 is frequently overexpressed in HNSCC, is important for cell proliferation, and sustains an undifferentiated state through activation of Rac1 and RhoA, followed by YAP activation [101]. Interestingly, VAV2 delays EGFR internalization and degradation, thus enhancing downstream signaling pathways [102]. Given that the EGFR-MOB1 axis leads to YAP activation, VAV2 overexpression may be involved in YAP activation in HNSCC by sustaining the EGFR-MOB1 and VAV2-RAC1-YAP axes (Figure 3).

**YAP-interacting molecules in the nucleus.** Emerging evidence suggests that YAP/TAZ/TEAD interacts with multiple factors in the nucleus. YAP/TEAD2 cooperates with transcription factor E2F and promotes cell cycle gene expression during relapse of KRAS-independent pancreatic ductal adenocarcinoma [103]. Activator protein-1 (AP-1; a dimer of JUN and FOS proteins) and YAP/TAZ/TEAD co-occupy the enhancer region of growth-related genes, resulting in synergistic cell proliferation [104]. YAP/TAZ binds to the promoter region of FOS and enhances its transcription, suggesting a positive feedback loop for AP-1 and YAP/TAZ [105]. The general co-activator bromodomain containing protein 4 (BRD4) physically interacts with YAP/TAZ to recruit YAP/TAZ-bound enhancers close to RNA polymerase II (POL II) at YAP/TAZ-regulated promoters, leading to enhanced transcription of growth-related genes [106]. YAP occupies enhancers or super-enhancers and recruits the mediator complex and the CDK9 elongating kinase, thereby releasing RNA POL II promoter pausing to boost transcriptional elongation [107]. The AT-rich interaction domain 1A (ARID1A)-containing SWI/SNF complex physically interacts with YAP/TAZ and suppresses YAP/TAZ-regulated genes [108]. Cyclin dependent kinase 7 (CDK7) phosphorylates YAP/TAZ at S128 and S90 to avoid ubiquitination for degradation by the CRL4^DCAF12^ E3 ubiquitin ligase complex [109]. Lysine-specific demethylase 1 (LSD1), a histone demethylase encoded by *KDM1A* gene, forms the nucleosome remodeling and deacetylase complex with YAP, enhancing the transcription of YAP-target genes [110]. Moreover, YAP/TAZ promotes polyamine levels and cell proliferation by activating transcription of ornithine decarboxylase 1 (Odc1), inducing the hypusination of eukaryotic translation factor 5A (eIF5A) to enhance translation of LSD1 [111]. Co-amplification and overexpression of the p53 family members *p63* and A*CTL6A*, encoding an *SWI/SNF* subunit, suppresses transcription of *WWC1* encoding KIBRA, in turn activating YAP [112] (Figure 3). Collectively, the understanding of the YAP/TAZ regulatory mechanism in the nucleus is expanding, which is also necessary for the advancement of precision medicine targeting YAP/TAZ.

**YAP-target genes involved in immune evasion.** The interaction between programmed cell death 1 (PD-1) in T cells and programmed cell death 1 ligand 1 (PD-L1) in tumor cells leads to T cell exhaustion. This immune checkpoint mechanism generates a new therapeutic approach that targets PD-1 or PD-L1. Emerging evidence suggests that YAP/TAZ are key regulators of PD-L1 expression in tumor cells. The transcriptional complex of activated YAP and TEAD binds to the PD-L1 promoter in EGFR-TKI-resistant lung adenocarcinoma cell lines [113]. YAP/TAZ binds to TEAD at the promoter region of PD-L1 in human breast cancer cell lines, which is not conserved in mouse cancer cell lines [114]. In contrast, YAP/TEAD binds to the enhancer region of PD-L1 in human non-small cell lung cancer (NSCLC) [115], human malignant pleural mesothelioma [116], and BRAF inhibitor-resistant melanoma cells [117]. In addition, BRD4 enrichment in both promoter and enhancer regions is important for PD-L1 transcription by YAP/TAZ/TEAD in HNSCC [118]. However, another study demonstrated that the loss of LATS1/2 triggers anti-tumor immune responses by releasing nucleic-acid-rich extracellular vesicles [119]. Although PD-L1 regulation by YAP/TAZ has been supported by many studies, further research is required (Figure 3).

## 4. Therapeutic Approach Targeting the Hippo Pathway and YAP/TAZ

**Figure 5:**
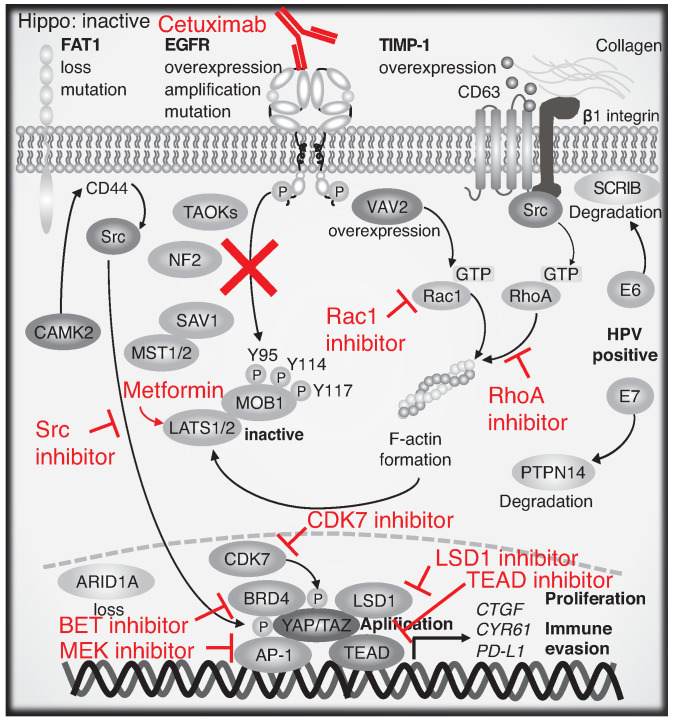
Schematic of therapeutic drugs targeting the Hippo pathway and YAP/TAZ in HNSCC. Metformin, Src, RhoA, Rac1, BET, LSD1, MEK, CDK7, and TEAD inhibitors are potential drugs for HNSCC treatment. AMPKa2 denotes AMP-activated catalytic subunit alpha 2 (also abbreviated AMPK).

Multiple drugs targeting the Hippo pathway and YAP/TAZ have been identified and examined. Here, we discuss representative therapeutic approaches and representative drugs that are undergoing clinical trials (Figure 5 and Table 1).

**Inhibitors targeting YAP or TEAD.** Although a few studies have demonstrated drugs that effectively target YAP, antisense oligonucleotides ION-537 reduce YAP protein levels, thereby suppressing tumor growth in a mouse model of hepatocellular cancer and a xenograft model of HNSCC cell lines harboring FAT1 mutation, which is in Phase I clinical trial [120] (Table 1).

Several approaches to target TEAD have been described. The first approach focuses on the nucleocytoplasmic shuttling of TEAD. Osmotic stress induces p38 activation, which binds to TEAD and induces its cytoplasmic translocation. Osmotic stress or p38 overexpression suppresses tumor growth in YAP-driven mesothelioma or uveal melanoma cells [121]. Thus, drugs that induce TEAD cytoplasmic localization can be a therapeutic option for patients with YAP-driven cancer (Figure 3 and Figure 5).

The second approach is to focus on vestigial-like (VGLL) proteins competing with YAP/TAZ for TEAD. Super-TDU, a peptide that mimics VGLL4, potently suppresses gastric tumor growth [122]. Moreover, a TEAD inhibitor in a mouse model, in which the Super-TDU peptide was modified to suppress both YAP-TEAD and TAZ-TEAD interactions, reportedly suppressed E2F transcription and cell proliferation and promoted differentiation through Kruppel-like factor 4 (KLF4) activation in keratinocytes [123]. Verteporfin, a photosensitizer used for the treatment of neovascular macular degeneration, was identified as the first drug that inhibits the YAP-TEAD interaction [124] (Figure 3).

The third approach is to focus on the YAP-binding site of TEAD. Several studies have identified a central pocket in the YAP-binding domain (YBD) of TEAD as a targetable site for small molecule inhibitors, including flufenamic acid, a non-steroidal anti-inflammatory drug (NSAID) [125,126]. Furthermore, TEADs have an intrinsic palmitoylating enzyme-like function, and the auto-palmitoylate cysteine of TEADs is essential for binding to YAP/TAZ [127]. During palmitoylation of TEAD, palmitate is attached via thioester linkage to a cysteine residue, which is supplied exogenously by diet or de novo biosynthesized by fatty acid synthase (FASN) [128]. DC-TEADin02, a potent and selective TEAD auto-methylation inhibitor, was identified in a biochemical study [129]. A small molecule library screen using a TEAD-dependent luciferase reporter identified MGH-CP1 as a TEAD auto-methylation inhibitor [130]. A small molecule that antagonistically binds to the TEAD YBD lipid pocket was shown to potently inhibit TEAD function [131]. VT103 and VT104 are compounds that prevent TEAD auto-methylation and inhibit tumor growth of mesothelioma [132]. MYF-01-37 binds covalently to TEAD and disrupts the YAP-TEAD interaction, enhances EGFR inhibitor-mediated apoptosis, and prevents dormancy in EGFR-mutant non-small cell lung cancer [133]. Currently, IK-930 is undergoing Phase I clinical trial [134] (Table 1). Therefore, targeting YAP itself or the YAP-TEAD interaction is a promising therapy for HNSCC patients (Figure 5).

**ROCK inhibitor.** TIMP-1, Src, and p53 DNA contact mutations and mechanotransduction hyperactivate RhoA/ROCK1/actomyosin signaling. This, in turn, promotes YAP/TAZ-driven carcinogenesis. ROCK inhibitor selectively antagonizes YAP/TAZ-promoted proliferation in cancer [135] (Figure 5). No clinical trials (solid tumors, unspecified or head and neck) are undergoing.

**Inhibitors targeting YAP-interacting molecules.** CDK7 selective inhibitor THZ1 reduces CDK7-mediated phosphorylation of YAP/TAZ, thereby inducing its degradation by the CRL4^DCAF12^ E3 ubiquitin ligase complex [109] (Figure 5). Several drugs (BTX-A51, fadraciclib, samuraciclib, and SY-5609) are in Phase I or II clinical trials (Table 1). The bromodomain and extra-terminal motif (BET) inhibitor JQ1 suppresses YAP/TAZ-driven gene expression and tumorigenesis [106] (Figure 5). Five drugs including BI-894999, BPI-23314, NUV-868, PLX-2853, and SYHA-1801 are in Phase I or II clinical trials (Table 1). LSD1 knockout reportedly suppressed 4NQO-driven mouse tongue oral squamous cell carcinoma and LSD1 inhibition by SP2509 inhibited 4NQO-driven oral squamous cell carcinoma in combination with verteporfin [110]. Inhibition of LSD1 by SP-2577 inhibited liver carcinogenesis in YAP transgenic mice and breast cancer cell growth in an in vivo xenograft model [111] (Figure 5). Three drugs including JBI-802, seclidemstat, and CC 90011 are in Phase II clinical trial (Table 1).

**MEK inhibitor.** AP-1 and YAP/TAZ synergistically promotes growth-related genes [104], and, given that upstream MAPK-extracellular signal-regulated kinase pathway is frequently activated in HNSCC by EGFR or other receptor tyrosine kinases (RTKs), MEK inhibitors including trametinib can be a potential drug targeting AP-1/YAP/TAZ-driven HNSCC progression. In addition, trametinib, an MAPK kinase (MEK) inhibitor, confers YAP overexpression and growth-related gene expression in HNSCC cell lines and patient-derived xenograft models, suggesting an effective combination therapy targeting YAP and MEK for HNSCC patients [136] (Figure 5). Four drugs including trametinib, binimetinib, cobimetinib, and mirdametinib are in Phase II clinical trials (Table 1).

**Src inhibitor.** Tyrosine phosphorylation of YAP is also important for its activity, as well as serine/threonine phosphorylation. Tyrosine 357 of YAP is phosphorylated by Yes, Src, and focal adhesion kinase (FAK) [137,138,139,140]. Moreover, Y341, Y357, and Y394 of YAP phosphorylation by Src enhances transcriptional activity, nuclear accumulation, and interaction with TEAD of YAP in skin squamous cell carcinoma [141]. Dasatinib, an Src family kinase inhibitor, suppressed the initiation of YAP1-induced tongue squamous cell carcinoma in a mouse model [44]. Therefore, Src inhibitors are promising drugs that target YAP (Figure 5). Five drugs including dasatinib, repotrectinib, VAL-201, TPX-0046, and elzovantinib are in Phase II or III clinical trials (Table 1).

**Metformin.** Energy stress is an important regulatory factor for YAP/TAZ. AMP-activated catalytic subunit alpha 2 (AMPKa2; also known as AMPK) activated by energy stress directly phosphorylates YAP Ser 94. This induces the dissociation of YAP-TEAD binding and indirectly inactivates YAP through LATS1/2 inactivation [142]. Metformin-induced energy stress also inactivates LATS1/2 through the inhibition of Rho GTPase and actin cytoskeleton dynamics as well as AMPK activation [143]. AMPK activated by energy stress phosphorylates and increases the protein stability of angiomotin like 2 (AMOTL2), which induces LATS1/2 activation and YAP inactivation [144]. Moreover, YAP is O-GlcNAcylated by O-GlcNAc transferase at serine 109 under high-glucose conditions, which prevents YAP inactivation by LATS1/2. Thus, low glucose levels induce YAP inactivation by loss of O-GlcNAcylation independent of AMPK activation [145]. Metformin, a drug used for patients with type 2 diabetes, as well as other AMPK activators, reportedly suppress tumorigenicity and YAP activity via AMPK activation in vitro and in vivo [142]. In addition, AMPK activation also suppresses mTOR signaling, which is important for premalignancy, initiation, and progression of HNSCC proved in past clinical trials [146,147,148,149,150] (Figure 5). Thus, metformin may prove to be a valuable precision drug.

## 5. Conclusions and Future Directions

Significant progress has been made in understanding the molecular and genetic mechanisms of the Hippo pathway and YAP/TAZ. Abundant evidence integrating genetic alterations in cancer with the Hippo pathway has emerged. The collective evidence will inform the development of drugs targeting the Hippo pathway specifically and, more generally, the future of precision medicine for HNSCC patients. Simultaneously, drug delivery systems, including antisense oligonucleotides, mRNA, liposomes, and nanoparticles, have dramatically advanced. These developments will aid in the realization of therapeutic approaches targeting the Hippo pathway and YAP/TAZ in cancer cells. Given that EGFR-targeted therapy resistance involves YAP/TAZ reactivation and that TEAD inhibitors may represent effective for YAP/TAZ signaling blockade, targeting the Hippo pathway and YAP/TAZ may be expanded toward combination therapy with other existing drugs, including tyrosine kinase inhibitors, antibodies, and immune checkpoint inhibitors. As the functions of the Hippo pathway and YAP/TAZ differ in many cancer types, it is possible cancer heterogeneity may underlie distinct roles for YAP/TAZ in different HNSCC subpopulations. Further studies, including single-cell approaches, will uncover the comprehensive and heterogeneous activity of YAP/TAZ in HNSCC.

Among the multiple drugs targeting the Hippo pathway, YAP/TAZ/TEAD and their regulators, TEAD inhibitors appear to emerge as the most effective and potent suppressors of YAP/TAZ-driven cancer growth, because TEADs bind YAP/TAZ directly and, hence, are the most downstream effectors of the pathway. Future studies will uncover the effect of TEAD inhibitor treatment alone and advanced combination therapies using TEAD inhibitors for HNSCC patients.

Collectively, integrating genetic alterations and the Hippo pathway has opened a new field of cancer research. This includes the development of analyses or drugs that will alter and improve clinical examination and treatment of HNSCC patients. Further studies will reduce a long distance between emerging biological evidence and current clinical trials and assist the development of precision medicine for HNSCC patients.

## Figures and Tables

**Table 1:** Representative potential drugs targeting the Hippo pathway undergoing clinical trials. Potential drugs undergoing clinical trials were searched using “head and neck cancer” or “solid, unspecified” terms using the Pharmaprojects intuitive interface of Citeline. Drugs shown as “Preclinical” were removed.

Drug Name	Company	Target	Clinical Trials (ClinicalTrials.gov Identifier)	Cancer Type
**IK-930**	Ikena Oncology	TEAD	Phase I (NCT05228015)	Solid, unspecified
**ION-537**	Ionis Pharmacueticals	YAP	Phase I (NCT04659096)	Solid, unspecified
**BTX-A51**	BioTheryX	CDK7	Phase I (NCT04872166)	Solid, unspecified
**fadraciclib**	Cyclacel	CDK7	Phase II (NCT04983810)	Solid, unspecified
**samuraciclib**	Carrick Therapeutics, Evotec	CDK7	Phase II (NCT03363893)	Unspecified
**SY-5609**	Syros Pharmaceuticals	CDK7	Phase I (NCT04247126)	Solid, unspecified
**XL-102**	Exelixis	CDK7	Phase I (NCT04726332)	Solid, unspecified
**trametinib**	Novartis	MEK	Phase II (NCT01376310)	Solid, unspecified
**binimetinib**	Pfizer	MEK	Phase II (NCT01885195)	Solid, unspecified
**cobimetinib**	Roche	MEK	Phase II (NCT02639546)	Solid, unspecified
**mirdametinib**	SpringWorks Therapeutics	MEK	Phase II (NCT05054374)	Solid, unspecified
**BI-894999**	Boehringer Ingelheim	BRD4	Phase I (NCT02516553)	Solid, unspecified
**BPI-23314**	Betta Pharmaceuticals	BRD4	Phase I (CTR20192223, China FDA)	Solid, unspecified
**NUV-868**	Nuvation Bio	BRD4	Phase II (NCT05252390)	Solid, unspecified
**PLX-2853**	Daiichi Sankyo	BRD4	Phase II (NCT03297424)	Solid, unspecified
**SYHA-1801**	CSPC Pharmaceutical	BRD4	Phase I (NCT04309968)	Solid, unspecified
**JBI-802**	Jubilant Life Sciences	LSD1	Phase II (NCT05268666)	Solid, unspecified
**seclidemstat**	Salarius Pharmaceuticals	LSD1	Phase II (NCT05266196)	Solid, unspecified
**CC 90011**	Bristol-Myers Squibb	LSD1	Phase II (NCT02875223)	Solid, unspecified
**dasatinib**	Bristol-Myers Squibb	Src	Phase II (NCT00882583)	Head and neck
**repotrectinib**	Turning Point Therapeutics, Zai Lab	Src	Phase III (NCT05004116)	Solid, unspecified
**VAL-201**	ValiRx	Src	Phase II (NCT02280317)	Solid, unspecified
**TPX-0046**	Turning Point Therapeutics	Src	Phase II (NCT04161391)	Solid, unspecified
**elzovantinib**	Turning Point Therapeutics	Src	Phase II (NCT03993873)	Solid, unspecified

## Data Availability

The landscape of the Figure 2 was created by c-BioPortal for Cancer Genomics (https://www.cbioportal.org accessed on 19 September 2022) by analyzing the data of the HNSCC TCGA, PanCancer Atlas (2015).
